# Neurologic manifestations of Long COVID in Colombia: a comparative analysis of post-hospitalization vs. non-hospitalized patients

**DOI:** 10.3389/fnhum.2024.1450110

**Published:** 2024-08-13

**Authors:** Carolina Hurtado, Diego Fernando Rojas-Gualdrón, Gina S. Pérez Giraldo, Esteban Villegas Arbelaez, Salvador Ernesto Medina Mantilla, Mariana Campuzano-Sierra, Santiago Ospina-Patino, Mariana Arroyave-Bustamante, Valeria Uribe-Vizcarra, Daniel Restrepo-Arbelaez, Paul Cardona, Julián Llano-Piedrahita, Santiago Vásquez-Builes, Esteban Agudelo-Quintero, Juliana Vélez-Arroyave, Sebastián Menges, Millenia Jimenez, Janet Miller, Yina M. Quique, Igor J. Koralnik

**Affiliations:** ^1^School of Medicine, CES University, Medellín, Colombia; ^2^Neurology Department, Medical University of South Carolina, Charleston, SC, United States; ^3^Clínica CES, Medellín, Colombia; ^4^The Ken & Ruth Davee Department of Neurology, Northwestern University Feinberg School of Medicine, Chicago, IL, United States; ^5^Shirley Ryan AbilityLab, Chicago, IL, United States

**Keywords:** COVID-19, post-acute COVID-19 syndrome, neurologic manifestations, cognitive dysfunction, patient reported outcome measures

## Abstract

**Objective:**

To analyze patient-reported outcomes, cognitive function, and persistent symptoms in patients with neurologic post-acute sequelae of SARS-CoV-2 infection (Neuro-PASC) in Colombia.

**Methods:**

We recruited patients with laboratory-confirmed COVID-19 and PASC symptoms lasting more than 6 weeks at the CES University and CES Clinic (Medellín, Colombia). We included 50 post-hospitalization Neuro-PASC (PNP) and 50 non-hospitalized Neuro-PASC (NNP) patients. Long-COVID symptoms, cognitive (NIH Toolbox v2.1-Spanish for 18+), patient-reported (PROMIS) outcomes, and relevant medical history were evaluated. Statistical analyses were performed via generalized linear models.

**Results:**

Overall, brain fog (60%), myalgia (42%), and numbness or tingling (41%) were the most common neurological symptoms, while fatigue (74%), sleep problems (46%), and anxiety (44%) were the most common non-neurological symptoms. Compared to NNP, PNP patients showed a higher frequency of abnormal neurological exam findings (64% vs. 42%, *p* = 0.028). Both groups had impaired quality of life (QoL) in domains of cognition, fatigue, anxiety depression and sleep disturbance, and performed worse on processing speed and attention than a normative population. In addition, NNP patients performed worse on executive function than PNP patients (T-score 42.6 vs. 48.5, *p* = 0.012). PASC symptoms of anxiety and depression were associated with worse QoL and cognitive outcomes. Brain fog and fatigue remained persistent symptoms across all durations of Long COVID.

**Conclusion:**

Our findings highlight the high incidence and heterogeneity of the neurologic symptoms and impacts of Long COVID even more than 2 years from disease onset. Early detection, emotional support and targeted management of Neuro-PASC patients are warranted.

## Graphical abstract

Designed with https://www.biorender.com/

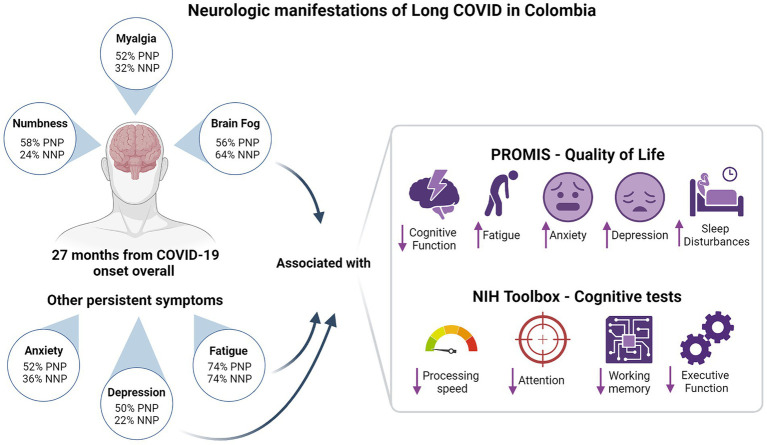

## Introduction

Long COVID or Post-Acute Sequelae of SARS-CoV-2 (PASC) is a broad term defined by the United States Centers for Disease Control and Prevention (CDC) as “signs, symptoms, and conditions that continue or develop after initial SARS-CoV-2 infection” that “are present 4 weeks or more after the initial phase of infection” (Affairs ASPA, 2023). The definition varies between agencies and organizations, particularly measurement periods, and even a systematic review established that among 291 identified studies, 82.2% used an author definition (64.1%) or did not provide a prespecified definition (18.1%) (Yang et al., 2024).

Despite differences in definition, the evidence about the burden of Long COVID is clear. For instance, a Bayesian meta-regression of 56 sources from 22 countries, including 1.2 million patients with symptomatic SARS-CoV-2 infection, estimated that 6.2% (95% UI 2.4–13.3%) still experienced at least one symptom 3 months after infection (Global Burden of Disease Long COVID Collaborators et al., 2022). A national Long COVID household pulse survey in the United States showed that 17.6% of all adults have ever experienced Long COVID (Long COVID-Household Pulse Survey-COVID-19, 2024). Beyond the persistence or appearance of signs and symptoms, Long COVID patients tend to experience impaired quality of life (QoL) (Vélez-Santamaría et al., 2023; Bota et al., 2024), financial losses due to changes in their economic activity and informal caregiving (Kwon et al., 2023; Leitner et al., 2024), and tend to require more healthcare resources, increasing medical costs (Castriotta et al., 2024). The impact of Long COVID on mortality is less clear, with some studies showing short-term trends (Mainous et al., 2021; DeVries et al., 2023).

Clinical presentation of Long COVID patients is highly heterogeneous. A thematic analysis of patients’ experiences with Long COVID, including a literature review and patient and clinician interviews, identified that the most frequent symptoms are related to neurocognitive and systemic domains, impacting the emotional and independence for daily living activities (Rofail et al., 2024). Different reviews have highlighted the frequency and impact of sleep disturbances, depression, post-traumatic stress, anxiety, cognitive impairment, sensory disturbances, headaches, and dysautonomia among psychiatric and neurological symptoms (Marchi et al., 2023; Navis, 2023). However, it has been described that clinical profiles differ according to patients’ comorbidities, demographics, and their experience with COVID-19, particularly disease severity and history of hospitalization (Marjenberg et al., 2023).

Evidence shows that the duration of Long COVID symptoms is higher among hospitalized than among non-hospitalized patients (Global Burden of Disease Long COVID Collaborators et al., 2022) and that they have more severe and disabling symptoms (López-López et al., 2023) despite presenting a similar core symptoms pattern (O’Mahoney et al., 2023). However, evidence of Latin-American patients and comparisons considering potential confounding factors are scarce. Understanding the specific characteristics and clinical presentation of Long COVID symptoms is crucial to implementing and adapting intervention strategies to reduce its impact on functionality and QoL.

This study aimed to analyze the patient-reported outcomes, cognitive outcomes, and PASC symptoms in Long COVID patients in Colombia with and without a history of hospitalization for COVID-19 and to explore their association with their medical history. Our study represents the critical need for research focusing on Latin-American patients, as the current literature on Long COVID is deficient in representing this demographic. By shedding light on regional variations in Long COVID symptoms prevalent among Colombian patients, our investigation addresses a significant gap in knowledge and offers insights crucial for interventions and healthcare strategies in this population.

## Methods

This report follows the STROBE statement for cohort studies (Von Elm et al., 2008). The CES University Institutional Review Board approved the study protocol (code 1099, session 214 of 2022). Written informed consent was obtained from all enrolled patients in accordance with the Declaration of Helsinki and Colombian regulations for human health research.

### Study design and settings

An ambispective cohort study was conducted at the CES University School of Medicine and the CES Clinic outpatient facilities (Medellín, Colombia). Patients diagnosed with COVID-19 between April 2020 and August 2023 were recruited and evaluated from April 2023 to December 2023. PASC symptoms were retrospectively determined during the study evaluation; abnormal findings, QoL, and cognitive performance at the time of the study visit were evaluated prospectively.

### Participants

We included patients with a history of COVID-19 confirmed by PCR, serology, or antigen test who presented PASC symptoms for 6 weeks or more after the resolution of the acute infection. Patients were excluded if they could not fulfill the self-reported outcomes and cognitive tests because of severe neurological compromise or inability to read. All consecutive patients who fulfilled the eligibility criteria were included.

Patients previously hospitalized for COVID-19 at the CES Clinic and community volunteers identified by social media campaigns (approved by the IRB) were invited to participate in the study.

### Variables and measurement

The patient-reported outcomes Cognitive function (SF v2.0-4a), Fatigue (SF v1.0-4a), Sleep disturbances (SF v1.0-4a), Anxiety (SF v1.0-4a), and Depression (SF v1.0-4a) measured by the PROMIS (Lai et al., 2011, 2014) QoL scales were the primary outcomes. As secondary outcomes, we defined the performance on the cognitive scales of the NIH Toolbox v2.1-Spanish for 18+ (Gershon et al., 2010, 2013; Weintraub et al., 2013; Heaton et al., 2014): Processing speed (Pattern Comparison Processing Speed Test, Age 7+), Attention (Flanker Inhibitory Control and Attention, Age 12+), Executive function (Dimensional Change Card Sort Test, Age 12+), and Working memory (Picture Sequence Memory Test, Age 8 + -Form A) from the NIH Toolbox. It is relevant to highlight that the Flanker test focuses more on inhibitory control, and the Dimensional card sort test focuses more on cognitive flexibility, but both imply attentional processes for optimal performance (Heaton et al., 2014).

Additionally, PASC symptoms determined by neurological examination were included as secondary outcomes. We define PASC symptoms as those that developed or exacerbated and did not resolve 6 weeks or more after the resolution of the acute infection. All outcomes were measured and analyzed considering the duration of the Long COVID symptoms (age at study visit minus the age at COVID-19 symptoms onset).

The primary exposure variable of interest was the history of hospitalization for COVID-19. Patients were classified as post-hospitalization Neuro-PASC (PNP) or non-hospitalized Neuro-PASC (NNP). Among the hospitalized, we characterized hospital stay (in days), intubation, encephalopathy, and encephalitis. Additional clinical and demographic characteristics were assessed for all patients: Abnormal findings in the neurological examination, neurological and non-neurological comorbidities, vaccination, sex, years of education, and age at COVID-19 symptoms onset, and COVID-19 severity classified according to the World Health Organization criteria (World Health Organization, 2021).

### Bias

Several actions were considered to guarantee data quality. Neurological examination and clinical interview were performed by seven third and fourth-year neurology residents, who were trained, standardized, and supervised by a senior neurologist. Cognitive Outcomes and PROMIS were scored and analyzed under the supervision of a senior clinical neuropsychologist. All data were registered on a prespecified database on REDCap. Two general practitioners performed data curation and validation under the supervision of the co-principal investigator.

### Study size

**S**ample size of 50 PNP and 50 NNP patients was established by protocol. This sample size was targeted to achieve a statistical power > 0.80 for unadjusted mean *T*-score differences ≥5 points on the Cognitive and PROMIS scales, assuming alpha = 0.05, reference mean of 50, and common standard deviation (SD) of 8.5. *Post-hoc* estimates determined statistical power > 0.80 for risk ratios (RR) ≤ 0.46 or RR ≥ 2.17, assuming a reference risk of 50% and alpha = 0.05.

### Quantitative variables

PROMIS QoL scales are presented in their original *T*-score with a mean of 50 and SD of 10. Low scores indicate deficits in the subjective impression of Cognitive function and objective measurement of processing speed, attention, executive function, and working memory. High scores indicate subjective alterations in fatigue, anxiety, depression, and sleep.

### Statistical methods

Descriptive statistics are presented for patient demographics, medical history, and abnormal findings during the neurological examination. The Chi-squared (or Exact test) and Mann–Whitney tests were used to analyze differences in categorical and quantitative variables between PNP and NNP patients.

The incidence of neurological symptoms and signs according to hospitalization for COVID-19 was analyzed using a generalized linear model (GLM) with Poisson distribution, identity function, and robust variance estimation. Observed Risk Differences (RD) between PNP and NNP patients with 95% confidence intervals (95%CI) and *p*-values are presented in a forest plot for each symptom and sign.

Means and SD are provided for PROMIS and cognitive measures. A one-sample t-test was performed for each outcome to evaluate the difference from the reference population mean value T-score = 50, and an independent samples t-test was performed to evaluate the difference between PNP and NNP patients. We present all descriptive data by groups according to hospitalization for COVID-19 in swarmplots, which jointly present the boxplot with the distribution of each variable.

The association of neurological symptoms and signs with the PROMIS QoL and cognitive outcomes was also analyzed using the GLM. Adjusted mean differences (mean diff.) with 95%CI are provided for each outcome to control for confounding effects. The multiple regression model was used to adjust by COVID-19 severity, abnormal findings during the neurological examination, sex, years of education, age at the onset of COVID-19 symptoms, and time with Long-COVID symptoms.

Additionally, we explored which neurological symptoms presented a risk that varied with the time they lived with Long-COVID, and which neurological symptoms persisted with high risk among patients with a higher perceived percentage of recovery. The LASSO model (Least Absolute Shrinkage and Selection Operator) was used for these exploratory analyses. As the difference in the risk of neurological symptoms for each additional month lived with Long-COVID was not constant and a linear assumption was not held, we employed second (time + time^2^) or third (time + time^2^ + time^3^) degree polynomials to find the best fitting curve, according to Akaike (AIC) and Bayesian (BIC) information criteria. Marginal frequency estimates of risks are presented for symptoms with statistically significant association along the range of time they lived with Long-COVID and perceived percentage of recovery.

Statistical significance was set at *p*-value <0.05. The statistical criteria for variable selection for multiple regression was a p-value <0.25 for unadjusted estimates. All statistical analyses were performed in Stata version 16.1 (College Station, TX).

## Results

### Participants

**Figure 1:**
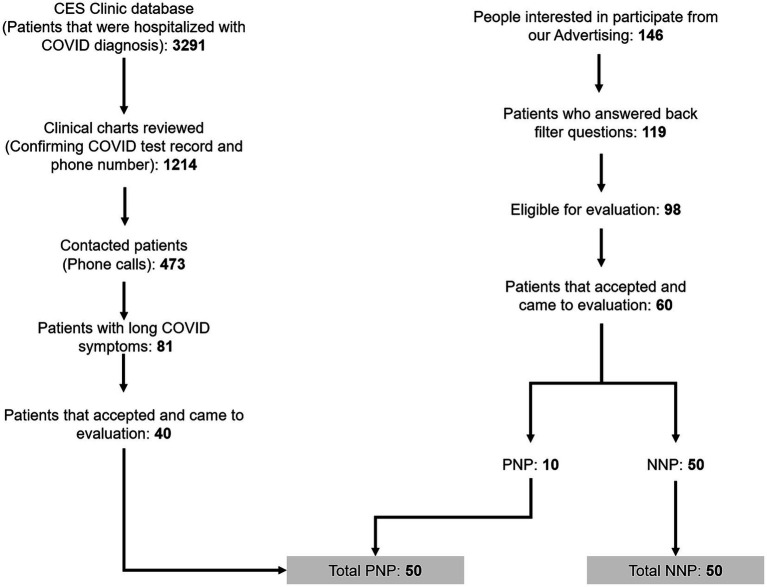
Patients’ recruitment process.

Figure 1 presents the study flow diagram; 1,214 patients with and 146 without a history of hospitalization for COVID-19 were considered potentially eligible for the study. Four hundred forty did not meet the inclusion criteria, and 340 were excluded because they did not come for evaluation. Finally, 50 PNP and 50 NNP patients were included and analyzed. Those patients provided data on 2577.4 months of Long COVID course, with a mean of 25.8 (SD 8.6) months.

### Descriptive data

#### Patients demographics and medical history

Table 1 presents descriptive data by history of hospitalization for COVID-19. PNP were older than NNP patients (51 vs. 36 years old, *p*-value <0.001), and there were fewer women among PNP patients (48% vs. 78%, p-value 0.002), and PNP had a lower median of years of education (13 vs. 16, p-value <0.001). Overall, patients were evaluated 27.4 months after COVID-19 symptoms onset, with no significant differences between PNP and NNP groups.

**Table 1:** Demographics and relevant medical history, by history of hospitalization for COVID-19.

	Overall (*n* = 100)	PNP (*n* = 50)	NNP (*n* = 50)	*p*-value
Age, Median (IQR)	43.5 [35–54.5]	51 (41–57)	36 (29–46)	**<0.001**
Female, *n* (%)	63 (63)	24 (48)	39 (78)	**0.002**
Race, Hispanic/Latino, *n* (%)	100 (100)	50 (100)	50 (100)	
BMI, Median (IQR)	27 [24–29.7]	28.9 (26.3–32.5)	24.7 (22.3–27.0)	**<0.001**
Years of education, Median (IQR)	16 [11–16]	13 (11–16)	16 (16–18)	**<0.001**
Months from symptoms onset, Median (IQR)	27.4 [21–30.4]	28.2 (24.4–30.4)	24.8 (17.1–31.9)	0.052
% recovery, Median (IQR)	70 [50–80]	70 (50–80)	70 (60–80)	1
Second infection, *n* (%)	20 (20)	4 (8)	16 (32)	**0.003**
SARS-CoV-2 vaccination, *n* (%)	92 (92)	44 (88)	48 (96)	0.134
SARS-CoV-2 infection after vaccination, *n* (%)	34 (34)	14 (28)	20 (40)	0.231
Hospitalization				
Hospital stay, Median (IQR)		23 (10–39)		
Intubation		20 (40)		
Encephalopathy		29 (58)		
Neurological comorbidities, *n* (%)				
Headache	28 (28)	12 (24)	16 (32)	0.373
Traumatic brain injury or concussion	1 (1)	1 (2)	0	
Encephalitis	1 (1)	1 (2)	0	
Non neurological comorbidities, *n* (%)				
Hypertension	23 (23)	16 (31)	7 (14)	**0.032**
Dyslipidemia	17 (17)	12 (24)	5 (10)	0.062
Diabetes, type II	9 (9)	9 (18)	0	**0.003**
COPD	4 (4)	4 (8)	0	
HIV	3 (3)	1 (2)	2 (4)	
Rheumatoid arthritis	2 (2)	1 (2)	1 (2)	
Coronary vascular disease	2 (2)	2 (4)	0	
Chronic kidney disease	2 (2)	2 (4)	0	
Smoking	2 (2)	2 (4)	0	
	Overall (*n* = 100)	PNP (*n* = 50)	NNP (*n* = 50)	*p*-value
Alcohol abuse	2 (2)	2 (4)	0	
Hashimoto	1 (1)	0	1 (2)	
Diabetes, type I	1 (1)	0	1 (2)	
Coronary artery disease	1 (1)	1 (1)	0	
Peripheral vascular disease	1 (1)	0 (0)	1 (2)	
Congestive heart failure	1 (1)	1 (2)	0	
Cancer	1 (1)	1 (2)	0	
Other drug abuse	1 (1)	1 (2)	0	

PNP, post-hospitalization Neuro-PASC; NNP, non-hospitalized Neuro-PASC; COPD, chronic obstructive pulmonary disease. Bold values highlights statistically different (*p*-value < 0.005).

In terms of relevant medical history, PNP patients had a higher median body mass index (28.9 vs. 24.7, *p*-value <0.001), a lower history of second infection (8% vs. 32%, *p*-value 0.003), and a higher prevalence of hypertension (31% vs. 14%, *p*-value 0.032) and diabetes type II (18% vs. 0%, p-value 0.003), compared to NNP patients. Additionally, several neurological and non-neurological comorbidities, listed in Table 1, were only observed among PNP patients. On the other hand, Hashimoto, Type I diabetes, and peripheral vascular disease were only observed among NNP, with one case each. The median hospital stay among PNP patients was 23 days (IQR 10–39). During the COVID-19 hospital stay, 40% of PNP patients required intubation, and 58% had encephalopathy.

### Outcome data

#### Neurologic and other PASC symptoms

**Figure 2:**
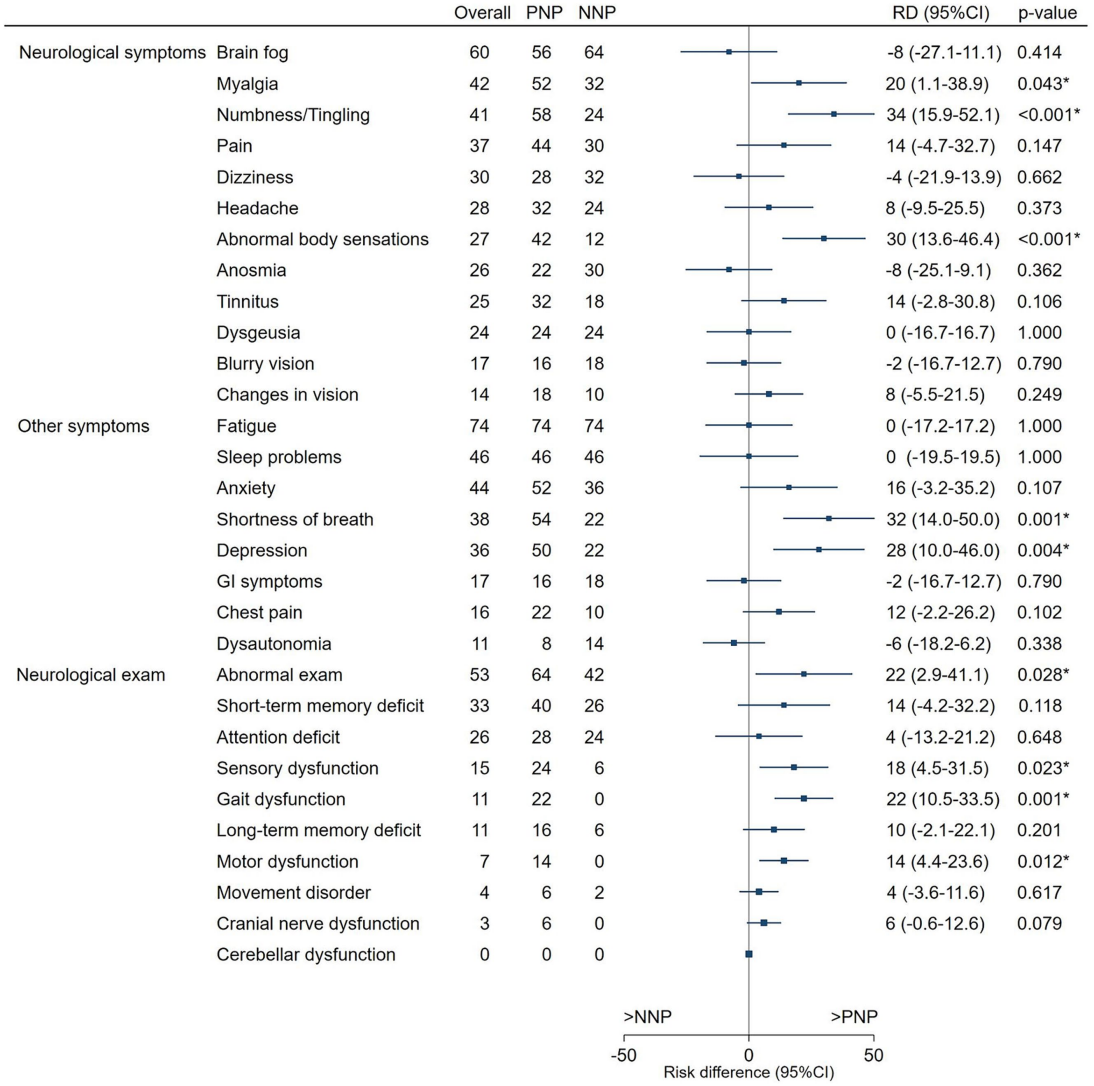
Neurologic symptoms and signs (%) by history of hospitalization for COVID-19. The forest plot shows the risk differences, with 95%CI and *p*-values, between PNP and NNP patients for neurologic and other symptoms and findings on the neurological exam. Symptoms and signs more frequently found in PNP patients are noted in positive values (right of the midline), and those more frequently found in NNP patients are in negative values (left of the midline). PNP, post-hospitalization Neuro-PASC; NNP, non-hospitalized Neuro-PASC; RD, risk difference; * highlights statistically different (*p*-value <0.005) risks between PNP and NNP.

The most frequent Neurologic symptoms overall were brain fog (60%), myalgias (42%), and numbness or tingling (41%), with numbness or tingling (58% vs. 24%, *p*-value <0.001) and “Abnormal body sensations” (42% vs. 12%, *p*-value <0.001) being more frequent in PNP than NNP patients. The most frequent non-neurologic symptoms overall were fatigue (74%), sleep problems (46%) and anxiety (44%) (Figure 2). PNP more frequently had shortness of breath (54% vs. 22%, *p*-value = 0.001) and depression (50% vs. 22%, *p*-value = 0.004) than NNP patients.

#### Alterations in the neurological exam

During the neurological exam, 64% of PNP patients presented abnormal findings compared to 42% of NNP patients (*p* = 0.028). A statistically significant difference was observed for sensory dysfunction (24% vs. 6%, *p*-value 0.023), gait dysfunction (22% vs. 0%, *p*-value 0.001), and motor dysfunction (14% vs. 0%, *p*-value 0.012) being more frequent in PNP patients (Figure 2).

### Quality of life measures

**Figure 3:**
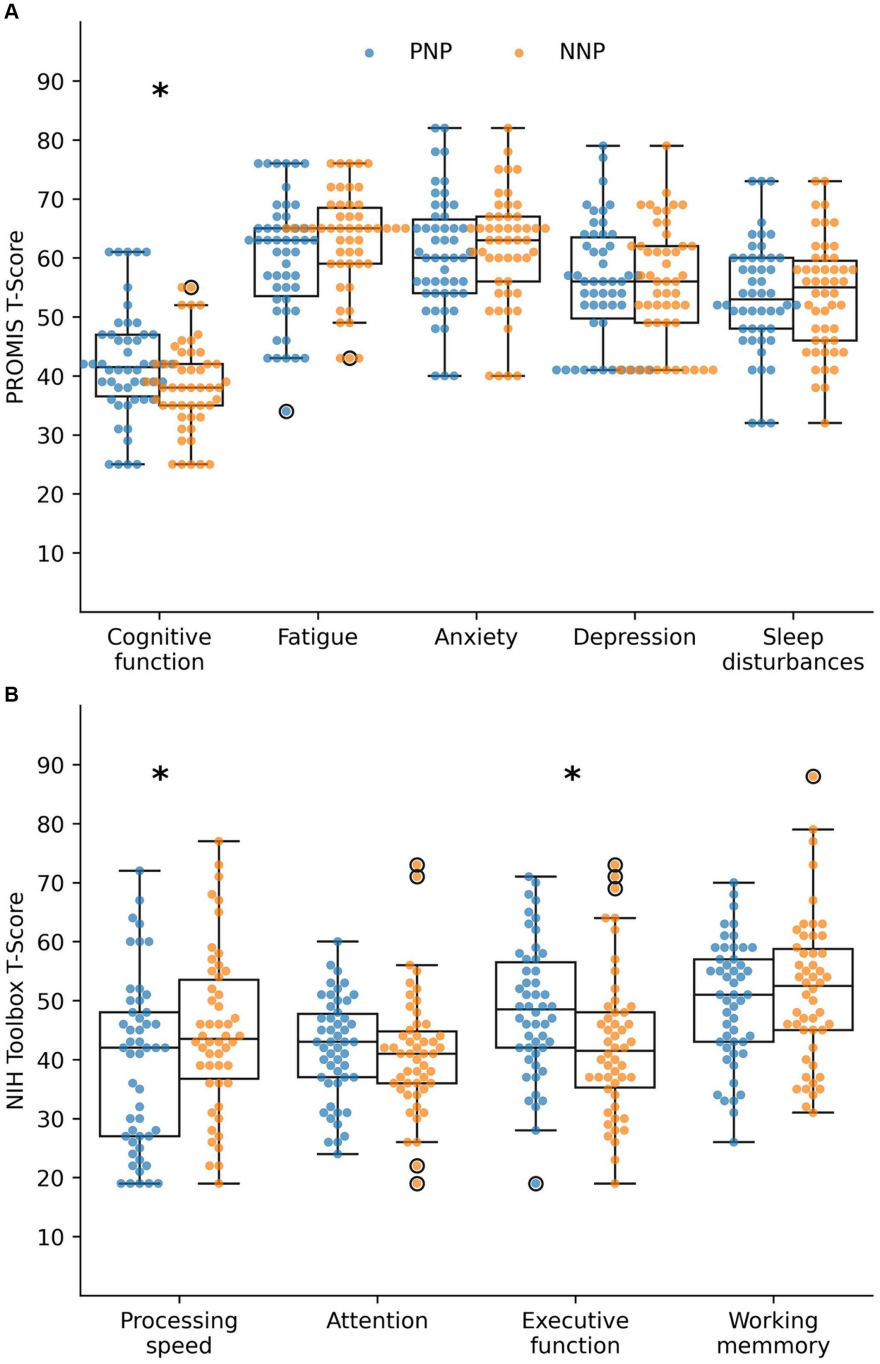
Quality of life **(A)** and cognitive outcomes **(B)** by history of hospitalization for COVID-19. Impact of Long COVID on the quality of life and cognition of PNP and NNP patients, measured by **(A)** PROMIS and **(B)** NIH Toolbox. A *T*-score of 50 indicates the mean of the reference population with a standard deviation of 10. Low scores indicate deficits in subjective impression of cognitive function, as well as in objective measurement of processing speed, attention, executive function, and working memory. High scores indicate subjective alterations in fatigue, anxiety, depression, and sleep. PNP, post-hospitalization Neuro-PASC; NNP, non-hospitalized Neuro-PASC. Bold *p*-value <0.05 for the null test mean = 50 (T-score). BG, Between groups PNP vs. NNP; QoL, quality of life. *Highlights statistically different (*p*-value < 0.005).

We analyzed the subjective QoL with the PROMIS measures, reported as *T*-scores (Figure 3). Among PNP patients, the most altered QoL domains were Anxiety, Fatigue, and Cognitive function, with mean *T*-scores of 60.7 (SD 9.9), 60.0 (SD 10.3), and 42.0 (SD 9.3), respectively, all in the mild impairment category (Table 2). These domains were also affected among NNP patients with mean *T*-scores of fatigue of 63.2 (SD 8.5), cognitive Function of 38.5 (SD 7.7), and anxiety of 61.4 (SD 9.6), all in the moderate impairment category (Table 2). All PROMIS mean values were statistically significantly worse than the reference mean for PNP and NNP patients, but only cognitive function was significantly worse in the NNP than in the PNP group (38.5 vs. 42.0, *p*-value 0.036).

**Table 2:** Descriptive statistics of the quality of life and cognitive outcomes by history of hospitalization for COVID-19.

	PNP		NNP		
	Mean (SD)	*p*-value norm	Mean (SD)	*p*-value norm	*p*-value BG
PROMIS - QoL					
Cognitive function	42.0 (9.3)	**<0.001**	38.5 (7.7)	**<0.001**	**0.036**
Fatigue	60.0 (10.3)	**<0.001**	63.2 (8.5)	**<0.001**	0.083
Anxiety	60.7 (9.9)	**<0.001**	61.4 (9.6)	**<0.001**	0.703
Depression	55.7 (10.2)	**0.001**	55.4 (10.1)	**0.001**	0.890
Sleep disturbance	53.5 (9.5)	**0.010**	53.7 (9.4)	**0.010**	0.890
NIH Toolbox					
Processing speed	39.3 (14.5)	**<0.001**	44.9 (13.6)	**0.007**	**0.047**
Attention	41.9 (8.6)	**<0.001**	41.1 (10.1)	**<0.001**	0.662
Executive function	48.5 (11.3)	0.400	42.6 (12.3)	**<0.001**	**0.012**
Working memory	49.8 (10.4)	0.961	51.8 (12.6)	0.575	0.380

PNP, post-hospitalization Neuro-PASC; NNP, non-hospitalized Neuro-PASC; *p*-value Norm, *p*-value for the test against the normative data (null hypothesis mean = 50); *P*-value BG, *p*-value for the mean difference between groups (null hypothesis PNP mean = NNP mean); QoL, Quality of Life. Bold. *p*-value < 0.05.

#### Cognitive outcomes

We then measured objective cognitive performance using the NIH Toolbox tests, reported as *T*-scores (Figure 3). Among PNP patients, Processing speed and attention were the most affected cognitive functions, with mean T-scores of 39.3 (SD 14.5) and 41.9 (SD 8.6), respectively, which were significantly worse than the reference population (Table 2). Among NNP patients, attention, Executive function, and Processing speed were the most compromised cognitive domains, with *T*-score means of 41.1 (SD 10.1), 42.6 (SD 12.3), and 44.9 (SD 13.6), respectively, which were significantly worse than the reference population. Processing speed was more affected in PNP than NNP patients (39.3 vs. 44.9, p-value 0.447), whereas executive function was more affected in NNP than in PNP patients (42.6 vs. 48.5, *p*-value 0.012) (Table 2).

### Main analysis

#### Association of PASC symptoms with PROMIS QoL and cognitive outcomes

**Figure 4:**
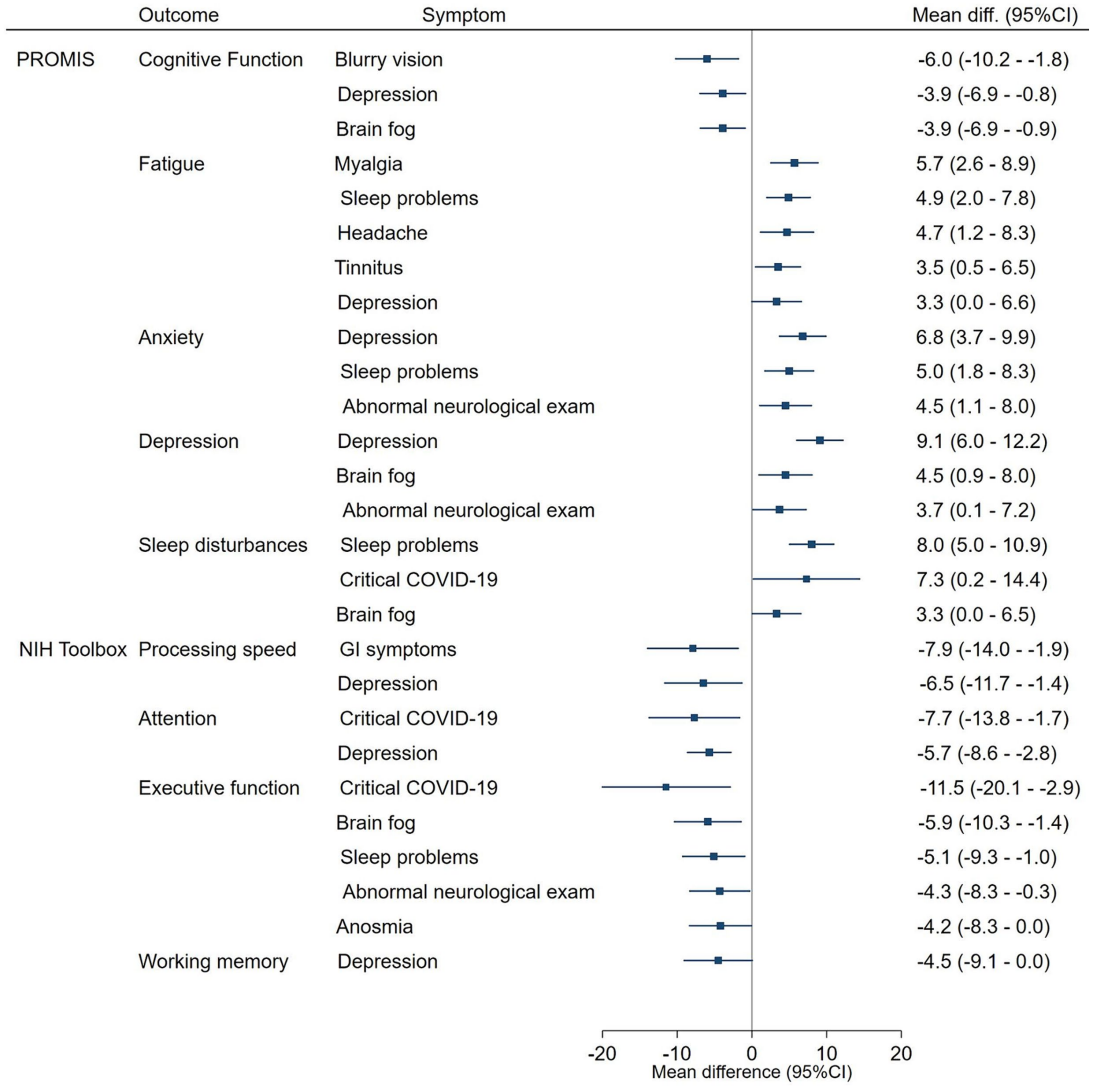
Neurologic symptoms and Signs associated with the PROMIS QoL measures and the NIH Toolbox cognitive tests. The forest plot shows the mean differences, with 95%CI and *p*-values, between patients with and without neurologic symptoms and signs in the PROMIS QoL and NIH toolbox cognitive tests. Only statistically significant differences are presented. Mean differences were adjusted by COVID-19 severity, abnormal findings during the neurological examination, sex, age at the onset of COVID-19 symptoms, and time with Long-COVID symptoms.

Figure 4 presents the neurological symptoms and signs that showed statistically significant adjusted association with each PROMIS and cognitive outcome on the overall sample.

Perceived fatigue was the PROMIS QoL domain with the highest number of PASC symptoms. Patients with myalgia (mean dif. 5.7, 95%CI 2.6–8.9), sleep problems (mean dif. 4.9, 95%CI 2.0–7.8), headache (mean dif. 4.7, 95%CI 1.2–8.3), tinnitus (mean dif. 3.5, 95%CI 0.5–6.5) and depression (mean dif. 3.3, 95%CI 0.0–6.6) had higher mean T-scores. Persistent sleep problems were also associated with higher mean T-scores on Anxiety (mean diff. 5.5, 95%CI 1.8–8.3), and perceived Sleep disturbances (mean diff. 8.0, 95%CI 5.0–10.9).

Conversely, Executive function was the Cognitive Outcome with the higher number of persistent symptoms: patients with Brain fog (mean diff. –5.9, 95%CI-10.3 – –1.4), Sleep problems (mean diff. –5.1, 95%CI-9.3 – –1.0), Abnormal findings on the neurological exam (mean diff. –4.3, 95%CI –8.3 – –0.3), Anosmia (mean diff. –4.2, 95%CI –8.3 – –0.0) had lower mean *T*-scores. Brain fog was also associated with lower perceived Cognitive function (mean diff. –3.9, 95%CI –6.9 – –0.9) and higher depression (mean diff. 4.5, 95%CI 0.9–8.0). Abnormal findings in the neurological exam were also associated with higher T-scores in Anxiety (mean diff. 4.5, 95%CI 1.1–8.0) and Depression (mean diff. 3.7, 95%CI 0.1–7.2).

PASC symptoms of depression were associated with six out of nine PROMIS and NIH Toolbox outcomes. Patients with those symptoms presented Lower Cognitive function (mean diff. –3.9, 95%CI –6.9 – –0.8), Processing Speed (mean diff. –6.5, 95%CI –11.7 – –1.4), Attention (mean diff. –5.7, 95%CI –8.6 – –2.8), and Working memory (mean diff. –4.5, 95%CI –9.1 –0.1), as well as higher perceived anxiety (mean diff. –5.7, 95%CI 3.7–9.9) and Depression (mean diff. 9.1, 95%CI 6.0–12.2).

Patients with a history of critical COVID-19 who required life-sustaining treatment showed lower *T*-scores on Executive function (mean diff. –11.5, 95%CI –20.1 – –2.9) and processing speed (mean diff. –7.7, 95%CI –13.8 – –1.7) and higher scores for Sleep disturbances (mean diff. 7.3, 95%CI 0.2–14.4).

### Other analyses

#### Frequency of symptoms according to time lived with Long COVID

**Figure 5:**
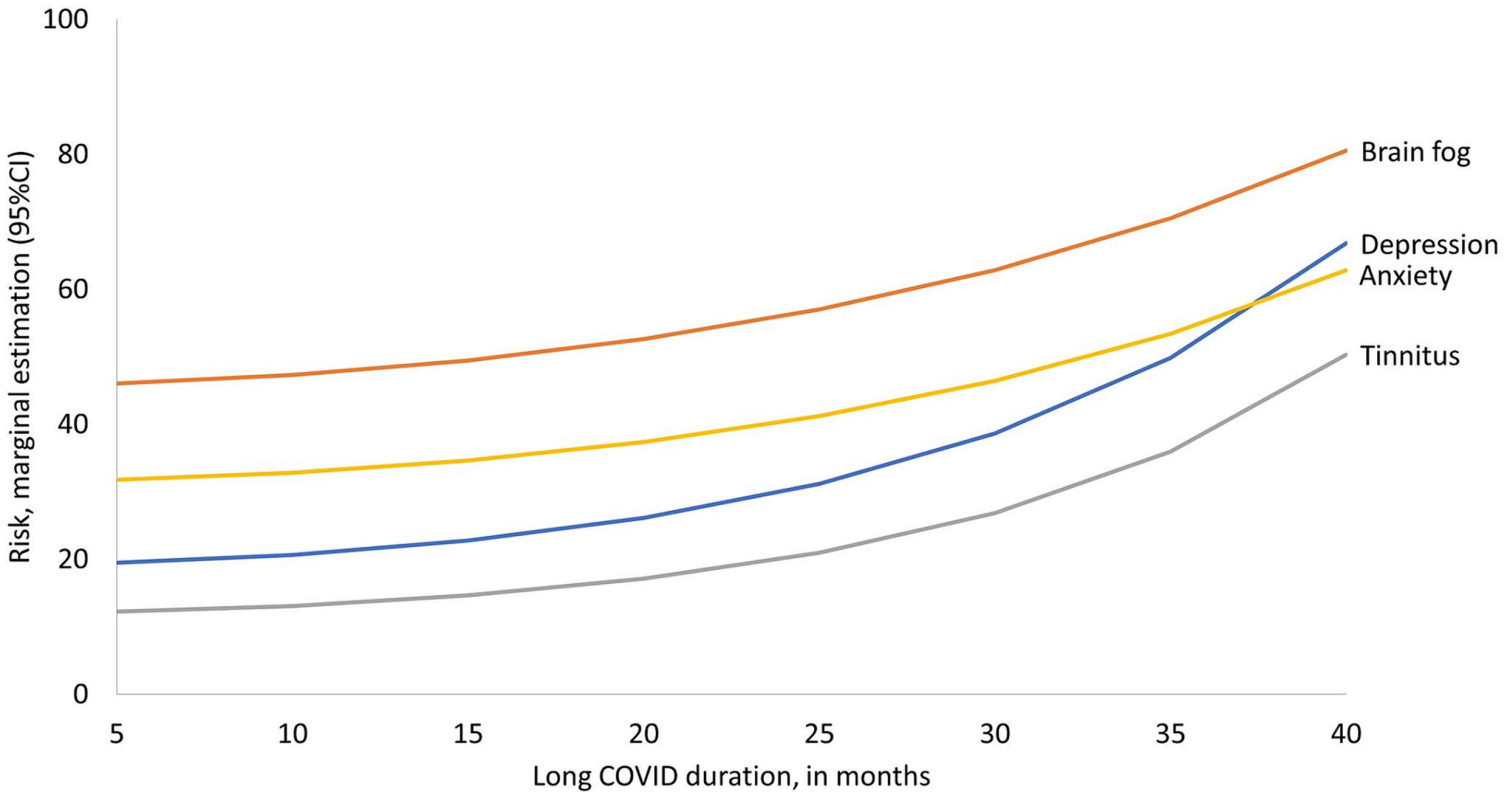
Frequency of symptoms according to time lived with Long COVID. The *X*-axis presents the observed values of time lived with Long COVID and the *Y*-axis presents the estimated risk of symptoms at each duration. Only symptoms with statistically significant association are presented.

We then analyzed the effect of time lived with Long COVID on symptom frequency among all PNP and NNP patients. According to the identified trend, brain fog, depression, anxiety, and tinnitus showed a slight increase per month lived with Long COVID, reflected in a slight slope among patients with 5–18 months post-COVID; among patients with more time lived with Long COVID the frequency of those symptoms pear each additional month increases which is reflected on steeper slopes (Figure 5). Depression and tinnitus exhibit a steeper slope, although brain fog is the most frequently reported symptom among patients with all durations. The average increases in those symptoms per each additional month of duration estimated by Lasso analyses were depression (RR = 1.54, tinnitus RR = 1.50, anxiety RR = 1.4), and brain fog RR = 1.22. There was no statistically significant association with the time spent with Long COVID for the remaining symptoms.

#### Frequency of symptoms according to perceived percentage of recovery

**Figure 6:**
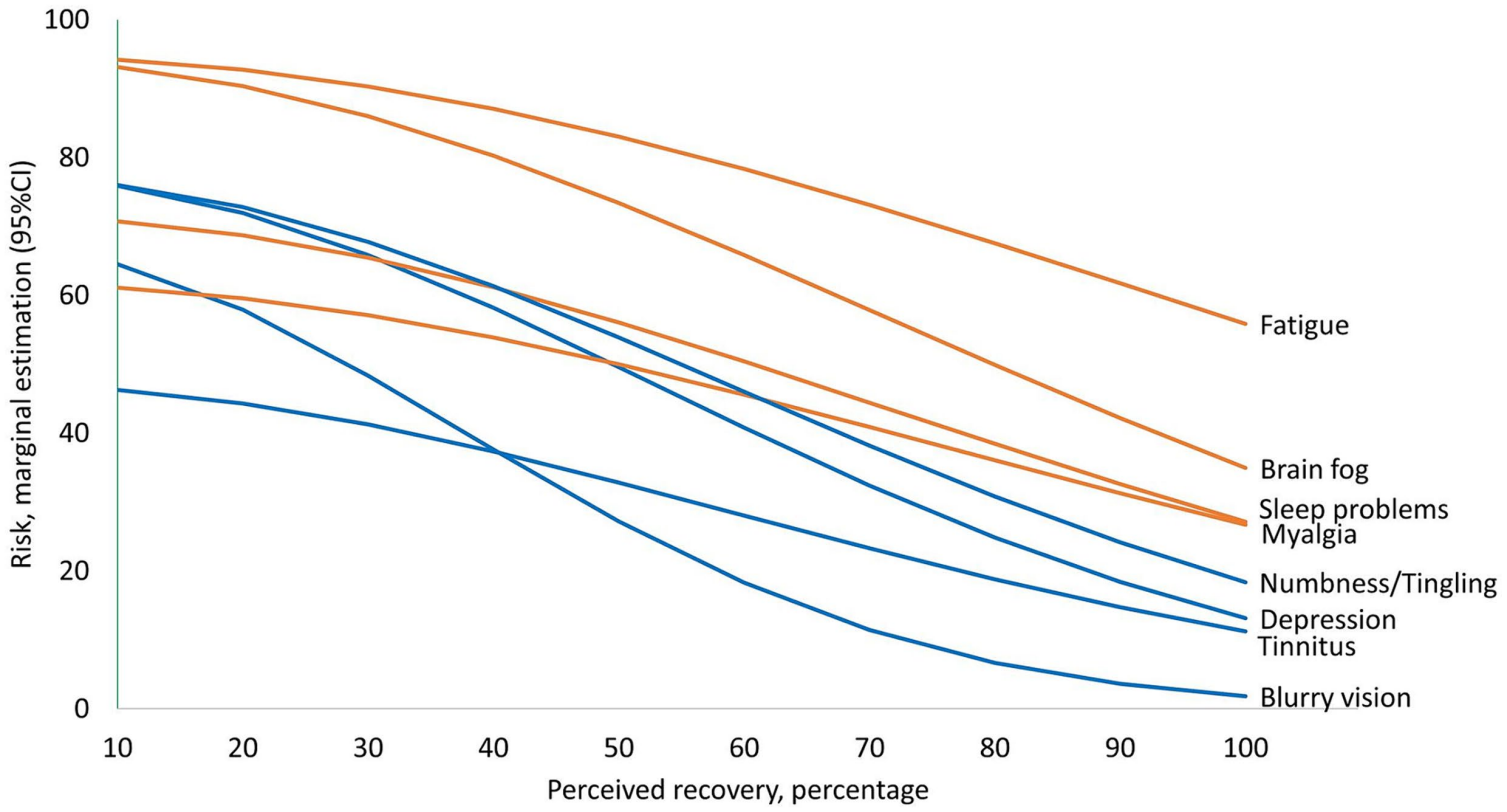
Frequency of symptoms according to perceived percentage of recovery. The *X*-axis presents the observed values of perceived percentage recovery, and the *Y*-axis presents the estimated risk of symptoms at each duration. Symptoms that tend to remain frequent even for patients with high perceived recovery are presented with orange lines. Symptoms markedly reduced among patients with higher perceived recovery are presented with blue lines. Only symptoms with statistically significant association are presented.

Two statistically significant trends were identified: symptoms that tend to remain frequent even for patients with high perceived recovery (Figure 6, orange lines) and symptoms markedly reduced among patients with higher perceived recovery (Figure 6, blue lines). Approximately 95% of patients with the lowest perceived percentage of recovery had Fatigue and Brain fog. Fatigue and brain fog are less frequent among patients with the highest perceived recovery, as presented in Figure 6 (orange lines), yet still around 1 in 2 patients experience fatigue, and 1 in 3 patients experience brain fog. On the other hand, the group of symptoms presented in Figure 6 with blue lines tended to reduce more drastically, with 47–77% of patients with the lowest perceived recovery experiencing them, compared to frequencies less than 1 in 4 to 1 in 10 patients among patients with the highest percentages of perceived recovery. The average difference in those symptoms per each 10% decrease in perceived recovery estimated by Lasso analyses were fatigue RR = 1.05, brain fog RR = 1.09, sleep problems RR = 1.10, myalgia RR = 1.10, numbness or tingling RR = 1.16, depression RR = 1.18, tinnitus RR = 1.11, and blurry vision RR = 1.33. There was no statistically significant association with the perceived percentage of recovery for the remaining symptoms.

## Discussion

This study aimed to analyze the patient-reported outcomes, cognitive outcomes, and persistent symptoms in Long COVID patients in Colombia. We found a high incidence of neurological and non-neurological persistent symptoms, with more than half of patients experiencing brain fog, fatigue, and abnormal neurological exam; objective evidence of cognitive dysfunction in processing speed, attention, and executive function; and patient-reported impact on cognitive function, fatigue, and anxiety. Executive function and perceived fatigue were the objective and subjective impacts associated with the most persistent symptoms. Numbness/tingling, myalgia, abnormal body sensations, shortness of breath, depression, and abnormal neurological exam were more frequently observed among PNP patients.

To the best of our knowledge, this is the first study to objectively evaluate and report an impairment in cognitive functions and a decrease in quality of life in patients with Long COVID in Colombia. Also, we describe the persistence of symptoms in a longer term of follow-up. Remarkably, our study shows that Long COVID symptoms can persist for years since our patients were evaluated 27 months from COVID-19 onset overall. Indeed, in the scarce evidence of cognitive impairment in other Latin American countries, patients were assessed with an overall of 142 days post-infection in Argentina (Crivelli et al., 2022), 6 months post-infection in Ecuador (Del Brutto et al., 2021), and 8 months post-infection in Brazil (Braga et al., 2022).

Our findings also add to the body of knowledge from North America, Europe and Asia highlighting the high incidence and heterogeneity of the symptoms and impacts experienced by Long COVID patients (Rofail et al., 2024). In particular, our study shows clearly that PNP and NNP groups have different demographics, comorbidities and symptoms. Indeed, NNP are 15 years younger than PNP patients, have a female predominance and have lower prevalence of pre-existing hypertension and type II diabetes. In addition, PNP and NNP patients have distinct symptomatology and neurologic examination findings. Indeed, PNP patients have higher prevalence of myalgia, numbness/tingling, and abnormal body sensations, as well as shortness of breath and depression. Furthermore, PNP patients more frequently have an abnormal neurologic exam, including sensory, motor and gait dysfunction. Moreover, PNP and NNP groups had different patterns of QoL alteration and cognitive dysfunction. Although both groups had worse QoL than the normative population in all 5 domains tested, NNP patients had worse subjective perception of their cognition than PNP patients. Finally, PNP and NNP patients have different patterns of cognitive dysfunction with PNP performing worse on processing speed test and NNP worse on executive function test. Altogether these particularities justify analyzing those two groups separately (Graham et al., 2021; Perez Giraldo et al., 2023). The differences in presentation and impact of Long COVID symptoms between patients with and without a history of hospitalization for COVID-19 have been reviewed by O’Mahoney et al. (2023) in a meta-analysis including 194 studies reporting data on 735,006 patients, showing that regardless of hospitalization status, 45% of patients were still experienced symptoms at 4 months.

To date, no classifications of “Long COVID severity” have been proposed, still our study, showed a wide range of impairment in persistent symptoms, quality of life, and cognitive test performance in both PNP and NNP patients. Particularly, the severity of Long COVID affecting NNP patients should not be underestimated, even though they only had a mild initial COVID-19 presentation. Indeed, previous studies that also evaluated the quality of life and cognitive tests at a Neuro-COVID-19clinic, in the US showed that NNP patients constitute most of the clinic population (Graham et al., 2021; Perez Giraldo et al., 2023).

A recent meta-analysis on the risk of Long COVID symptoms by Marjenberg et al. (2023) identified a pooled estimation of three times the risk of memory problems and two times the risk of concentration problems among Long COVID patients compared to non-infected controls; in our study, we found that 33 and 26% of Long COVID patients experienced short term memory and attention deficits, respectively. According to the review by Navis (2023), besides cognitive changes, other neurological symptoms such as sensory disturbances, headaches, and dysautonomia are also commonly experienced by Long COVID patients. In our study, those symptoms were experienced by 15, 28, and 11% of patients, respectively.

Psychiatric symptoms were also common among our patients, with incidences higher than average estimations reported in the literature. The systematic review of psychiatric symptoms in Long-COVID patients by Marchi et al. (2023) estimated weighted mean prevalences of 21.2% for depression, 15.8% for anxiety, 27.0% for sleep disturbances, compared with our findings of 36, 44, and 46%, respectively. The study by Taquet et al., one of the largest retrospective study reported 2-year incidences of mood disorder and anxiety of 11.5 and 18.8% and an incidence any psychiatric or neurological sequelae of 29.1% (Taquet et al., 2022). According to our findings, depression, and anxiety, in addition to being persistent post-COVID-19 symptoms, have a higher incidence among patients who have lived more time with Long COVID.

Fatigue (74%) and brain fog (60%) were the symptoms most frequently found in our study. In the meta-analysis of Di Gennaro et al. (2023), the pooled incidence of fatigue was 31.4%, Davis et al. reported 86.7% (Davis et al., 2021) and according to Marjenberg et al. (2023), the risk of fatigue among Long COVID patients is 72% higher compared to non-infected controls. Moreover, in our study, fatigue and brain fog were the symptoms that persisted even among patients with higher perceived percentages of Long COVID recovery. Brain fog has received particular attention as a relevant symptom among Long COVID patients, mainly because it appears to play a mediation role in the relationship between objective and subjective cognitive outcomes (Aghajani Mir, 2023; Delgado-Alonso et al., 2023). In addition there is evidence of abnormal resting-state EEG rhythms among Long COVID patients with brain fog who do not have pre-existing cognitive or affective disorders (Babiloni et al., 2024).

The reason why NNP patients are so severely affected by Long COVID is still unknown. Indeed, those individuals who never suffered from pneumonia and hypoxemia, have broad alteration of QoL and cognitive dysfunction, which have also been reported in previous studies of asymptomatic and mild COVID-19 patients (Adler et al., 2022; Bostanci et al., 2023). In our study, NNP patients presented with alterations in subjective cognitive function and objective measures of processing speed, attention and executive function compared to a normative population. Executive dysfunction has a relevant impact on daily life activities, which may explain the lower perceived cognitive function among NNP patients (Becker et al., 2023). Alternatively, NNP patients’ perception of cognitive dysfunction may be explained by the anguish associated with disabling Long COVID symptoms that seem out of proportion compared to having experienced asymptomatic or mild COVID-19 (Goodman et al., 2023). These finding highlight the need for the concomitant management of mental health in parallel to cognitive issues.

Regarding possible mechanisms that explain the cognitive alterations observed in patients with PASC, Toniolo et al. suggested a hypothesis of damage targeted to the frontal lobes or frontal networks (Toniolo et al., 2021). They also propose three possible underlying mechanisms: cytokine-mediated neurotoxicity, an autoimmune process, or viral invasion of the CNS through the olfactory bulb. One of the observations that support the theory of an autoimmune mechanism is the female predominance in patients with PASC (Perez Giraldo et al., 2023), which we also found in our study with 63% women. Furthermore, some studies have reported the presence of autoantibodies associated with SLE in patients with Long COVID, including patients with mild COVID (Visvabharathy et al., 2023) which could be a biological base of why non-hospitalized patients have severe symptoms during PASC. Furthermore, a recent preprint report on the presence of autoantibodies directed against the central nervous system, which, when transferred to mice, triggered a loss of balance and coordination (Santos Guedes De Sa et al., 2024).

This study has some limitations. The sample size was targeted to achieve enough statistical power for mean differences in PROMIS and cognitive outcomes ≥5 points (*T*-score), equivalent to half a standard deviation in the range of minimal important change for PROMIS measures (Terwee et al., 2021). However, for Long COVID symptoms, this sample size only provided enough statistical power for RR ≤ 0.46 or RR ≥ 2.17, which was statistically underpowered for potentially meaningful differences that we found for pain, tinnitus, anxiety, and short and long term memory deficits. Further studies with larger and more heterogeneous samples are required to verify the clinical and statistical significance of differences in those symptoms.

We did not evaluate the severity of symptoms, which, according to O’Mahoney et al. (2023), could be a relevant dimension for explaining differences between PNP and NNP patients. Including clinical scores for symptom severity in further studies with longer follow-up times may help better understand long-term prognosis. However, our study identified that the most persistent PASC symptoms, which do not resolve in the first months, have a detrimental QoL and cognitive impact and are associated with a lower percentage of perceived recovery. Future studies must understand how interactions between quantity, heterogeneity, severity, and persistence of PASC symptoms impact the patient’s QoL and functioning.

Due to budget constraints, we reconstructed the history of PASC symptoms during the study evaluation, but we did not include laboratory or neuroimaging to complement the neurological exam. No basal information was available for the QoL and cognitive outcomes, and reconstruction was not possible. Scoring individual patient performance against normative data from a reference population allows for identifying deficits but does not necessarily imply a decline in functioning. On the other hand, normative correction allows us to compare QoL and cognitive outcomes against the general population, but for PASC symptoms, we do not have uninfected controls for comparison.

## Conclusion

Colombia was one of the most affected countries in Latin America by the number of deaths from COVID-19, together with Brazil, Argentina, and Mexico. By extrapolating the prevalence of Long COVID observed in other regions, it is expected that approximately 29 million people in Latin America will suffer from Long COVID (Sakhamuri et al., 2022). The literature on Long COVID in Colombia is scarce and limited to describing the most frequent persistent symptoms, such as fatigue, sleep and memory problems, myalgias, and arthralgias (Anaya et al., 2021; Alvarez-Moreno et al., 2023; Angarita-Fonseca et al., 2023; Romero et al., 2023). However, there is a lack of evidence of objective measures of sequelae in our population. To the best of our knowledge, this is the first study of impairment in cognitive functions and, a decrease in quality of life in patients with Long COVID in Colombia. It also adds to a medium-term (27 months on average) description of persistent symptoms in Long COVID and their functional and cognitive impact.

Finally, our population of patients had symptoms that lasted for more than 2 years overall. Our exploratory analysis showed that the longer the duration of Long COVID, the higher the cumulative risk for persistent brain fog, depression and anxiety. Our results highlight the need for timely detection and early rehabilitation targeting modifiable symptoms, with an emphasis on brain fog, fatigue, and depression because of their detrimental impact on the quality of life and cognitive functions of our patients.

## Data Availability

The datasets presented in this article are not readily available because anonymized data, not published in the article, will be shared on reasonable request from a qualified investigator. Requests to access the datasets should be directed to churtadom@ces.edu.co.

## References

[ref1] Adler L. Gazit S. Pinto Y. Perez G. Mizrahi Reuveni M. Yehoshua I.. (2022). Long-COVID in patients with a history of mild or asymptomatic SARS-CoV-2 infection: a Nationwide cohort study. Scand. J. Prim. Health Care 40, 342–349. doi: 10.1080/02813432.2022.2139480, PMID: 36314555 PMC9848375

[ref2] Affairs ASPA. (2023). About Long COVID. Available at: https://www.covid.gov/be-informed/longcovid/about (Accessed February 6, 2024).

[ref3] Aghajani Mir M. (2023). Brain fog: a narrative review of the Most common mysterious cognitive disorder in COVID-19. Mol. Neurobiol. doi: 10.1007/s12035-023-03715-y [Epub ahead of print]., PMID: 37874482

[ref4] Alvarez-Moreno C. A. Pineda J. Bareño A. Espitia R. Rengifo P. (2023). Long COVID-19 in Latin America: Low prevalence, high resilience or low surveillance and difficulties accessing health care? Travel Med. Infect. Dis. 51:102492. doi: 10.1016/j.tmaid.2022.102492, PMID: 36368518 PMC9640373

[ref5] Anaya J.-M. Rojas M. Salinas M. L. Rodríguez Y. Roa G. Lozano M.. (2021). Post-COVID syndrome. A case series and comprehensive review. Autoimmun. Rev. 20:102947. doi: 10.1016/j.autrev.2021.102947, PMID: 34509649 PMC8428988

[ref6] Angarita-Fonseca A. Torres-Castro R. Benavides-Cordoba V. Chero S. Morales-Satán M. Hernández-López B.. (2023). Exploring long COVID condition in Latin America: its impact on patients’ activities and associated healthcare use. Front. Med. 10:1168628. doi: 10.3389/fmed.2023.1168628 PMC10157152 37153089

[ref7] Babiloni C. Gentilini Cacciola E. Tucci F. Vassalini P. Chilovi A. Jakhar D.. (2024). Resting-state EEG rhythms are abnormal in post COVID-19 patients with brain fog without cognitive and affective disorders. Clin. Neurophysiol. 161, 159–172. doi: 10.1016/j.clinph.2024.02.034, PMID: 38492271

[ref8] Becker J. H. Lin J. J. Twumasi A. Goswami R. Carnavali F. Stone K.. (2023). Greater executive dysfunction in patients post-COVID-19 compared to those not infected. Brain Behav. Immun. 114, 111–117. doi: 10.1016/j.bbi.2023.08.014, PMID: 37586567

[ref9] Bostanci A. Gazi U. Tosun O. Suer K. Unal Evren E. Evren H.. (2023). Long-COVID-19 in asymptomatic, non-hospitalized, and hospitalized populations: a cross-sectional study. JCM 12:2613. doi: 10.3390/jcm12072613, PMID: 37048697 PMC10095523

[ref10] Bota A. V. Bratosin F. Bogdan I. Septimiu-Radu S. Ilie A. C. Burtic S.-R.. (2024). Assessing the quality of life, coping strategies, anxiety and depression levels in patients with long-COVID-19 syndrome: a six-month follow-up study. Diseases 12:21. doi: 10.3390/diseases12010021, PMID: 38248372 PMC10814582

[ref11] Braga L. W. Oliveira S. B. Moreira A. S. Pereira M. E. Carneiro V. S. Serio A. S.. (2022). Neuropsychological manifestations of long COVID in hospitalized and non-hospitalized Brazilian patients. NRE 50, 391–400. doi: 10.3233/NRE-228020, PMID: 35599507

[ref12] Castriotta L. Onder G. Rosolen V. Beorchia Y. Fanizza C. Bellini B.. (2024). Examining potential long COVID effects through utilization of healthcare resources: a retrospective, population-based, matched cohort study comparing individuals with and without prior SARS-CoV-2 infection. Eur. J. Public. Health. 34, 592–599. doi: 10.1093/eurpub/ckae001, PMID: 38243748 PMC11161167

[ref13] Crivelli L. Calandri I. Corvalán N. Carello M. A. Keller G. Martínez C.. (2022). Cognitive consequences of COVID-19: results of a cohort study from South America. Arq. Neuropsiquiatr. 80, 240–247. doi: 10.1590/0004-282x-anp-2021-0320, PMID: 34816972 PMC9648931

[ref14] Davis H. E. Assaf G. S. McCorkell L. Wei H. Low R. J. Re’em Y.. (2021). Characterizing long COVID in an international cohort: 7 months of symptoms and their impact. eClinicalMedicine 38:101019. doi: 10.1016/j.eclinm.2021.101019, PMID: 34308300 PMC8280690

[ref16] Del Brutto O. H. Wu S. Mera R. M. Costa A. F. Recalde B. Y. Issa N. P. (2021). Cognitive decline among individuals with history of mild symptomatic SARS-CoV-2 infection: a longitudinal prospective study nested to a population cohort. Eur. J. Neurol. 28, 3245–3253. doi: 10.1111/ene.14775, PMID: 33576150 PMC8014083

[ref17] Delgado-Alonso C. Díez-Cirarda M. Pagán J. Pérez-Izquierdo C. Oliver-Mas S. Fernández-Romero L.. (2023). Unraveling brain fog in post-COVID syndrome: relationship between subjective cognitive complaints and cognitive function, fatigue, and neuropsychiatric symptoms. *Euro J of*. Euro J. Neurol.:ene.16084. doi: 10.1111/ene.16084 [Epub ahead of print]. PMC11618112 37797297

[ref18] DeVries A. Shambhu S. Sloop S. Overhage J. M. (2023). One-year adverse outcomes among US adults with post–COVID-19 condition vs those without COVID-19 in a large commercial insurance database. JAMA Health Forum 4:e230010. doi: 10.1001/jamahealthforum.2023.0010, PMID: 36867420 PMC9984976

[ref19] Di Gennaro F. Belati A. Tulone O. Diella L. Fiore Bavaro D. Bonica R.. (2023). Incidence of long COVID-19 in people with previous SARS-Cov2 infection: a systematic review and meta-analysis of 120, 970 patients. Intern. Emerg. Med. 18, 1573–1581. doi: 10.1007/s11739-022-03164-w, PMID: 36449260 PMC9709360

[ref20] Gershon R. C. Cella D. Fox N. A. Havlik R. J. Hendrie H. C. Wagster M. V. (2010). Assessment of neurological and behavioural function: the NIH toolbox. Lancet Neurol. 9, 138–139. doi: 10.1016/S1474-4422(09)70335-7, PMID: 20129161

[ref21] Gershon R. C. Wagster M. V. Hendrie H. C. Fox N. A. Cook K. F. Nowinski C. J. (2013). NIH toolbox for assessment of neurological and behavioral function. Neurology 80, S2–S6. doi: 10.1212/WNL.0b013e3182872e5f, PMID: 23479538 PMC3662335

[ref22] Global Burden of Disease Long COVID Collaborators Wulf Hanson S. Abbafati C. Aerts J. G. Al-Aly Z. Ashbaugh C.. (2022). Estimated global proportions of individuals with persistent fatigue, cognitive, and respiratory symptom clusters following symptomatic COVID-19 in 2020 and 2021. JAMA 328:1604. doi: 10.1001/jama.2022.18931, PMID: 36215063 PMC9552043

[ref23] Goodman M. L. Molldrem S. Elliott A. Robertson D. Keiser P. (2023). Long COVID and mental health correlates: a new chronic condition fits existing patterns. Health Psychol. Behav. Med. 11:2164498. doi: 10.1080/21642850.2022.2164498 36643576 PMC9833408

[ref24] Graham E. L. Clark J. R. Orban Z. S. Lim P. H. Szymanski A. L. Taylor C.. (2021). Persistent neurologic symptoms and cognitive dysfunction in non-hospitalized Covid-19 “long haulers.”. Ann. Clin. Transl. Neurol. 8, 1073–1085. doi: 10.1002/acn3.51350, PMID: 33755344 PMC8108421

[ref25] Heaton R. K. Akshoomoff N. Tulsky D. Mungas D. Weintraub S. Dikmen S.. (2014). Reliability and validity of composite scores from the NIH toolbox cognition battery in adults. J. Int. Neuropsychol. Soc. 20, 588–598. doi: 10.1017/S1355617714000241, PMID: 24960398 PMC4103963

[ref26] Kwon J. Milne R. Rayner C. Rocha Lawrence R. Mullard J. Mir G.. (2023). Impact of long COVID on productivity and informal caregiving. Eur. J. Health Econ. doi: 10.1007/s10198-023-01653-z [Epub ahead of print]., PMID: 38146040 PMC11377524

[ref27] Lai J.-S. Cella D. Choi S. Junghaenel D. U. Christodoulou C. Gershon R.. (2011). How item banks and their application can influence measurement practice in rehabilitation medicine: a PROMIS fatigue item Bank example. Arch. Phys. Med. Rehabil. 92, S20–S27. doi: 10.1016/j.apmr.2010.08.033, PMID: 21958919 PMC3696589

[ref28] Lai J.-S. Wagner L. I. Jacobsen P. B. Cella D. (2014). Self-reported cognitive concerns and abilities: two sides of one coin?: cognitive concerns versus cognitive abilities. Psycho-Oncology 23, 1133–1141. doi: 10.1002/pon.3522, PMID: 24700645 PMC4185008

[ref29] Leitner M. Pötz G. Berger M. Fellner M. Spat S. Koini M. (2024). Characteristics and burden of acute COVID-19 and long-COVID: demographic, physical, mental health, and economic perspectives. PLoS One 19:e0297207. doi: 10.1371/journal.pone.0297207, PMID: 38252638 PMC10802963

[ref30] Long COVID-Household Pulse Survey-COVID-19 (2024). Available at: https://www.cdc.gov/nchs/covid19/pulse/long-covid.htm (Accessed February 28, 2024).

[ref31] López-López L. Calvache-Mateo A. Ortiz-Rubio A. Granados-Santiago M. Heredia-Ciuró A. Martín-Núñez J.. (2023). Differences of disabling symptoms between previously hospitalized or non-hospitalized currently working long-COVID survivors one year after infection: a descriptive study. Healthcare 11:2306. doi: 10.3390/healthcare11162306, PMID: 37628505 PMC10454028

[ref32] Mainous A. G. Rooks B. J. Wu V. Orlando F. A. (2021). COVID-19 post-acute sequelae among adults: 12 month mortality risk. Front. Med. 8:778434. doi: 10.3389/fmed.2021.778434, PMID: 34926521 PMC8671141

[ref33] Marchi M. Grenzi P. Serafini V. Capoccia F. Rossi F. Marrino P.. (2023). Psychiatric symptoms in long-COVID patients: a systematic review. Front. Psych. 14:1138389. doi: 10.3389/fpsyt.2023.1138389 PMC10320160 37415689

[ref34] Marjenberg Z. Leng S. Tascini C. Garg M. Misso K. El Guerche Seblain C.. (2023). Risk of long COVID main symptoms after SARS-CoV-2 infection: a systematic review and meta-analysis. Sci. Rep. 13:15332. doi: 10.1038/s41598-023-42321-9 37714919 PMC10504382

[ref35] Navis A. (2023). A review of neurological symptoms in long COVID and clinical management. Semin. Neurol. 43, 286–296. doi: 10.1055/s-0043-1767781, PMID: 37068519

[ref36] O’Mahoney L. L. Routen A. Gillies C. Ekezie W. Welford A. Zhang A.. (2023). The prevalence and long-term health effects of long Covid among hospitalised and non-hospitalised populations: a systematic review and meta-analysis. eClinicalMedicine. 55:101762. doi: 10.1016/j.eclinm.2022.101762, PMID: 36474804 PMC9714474

[ref38] Perez Giraldo G. S. Ali S. T. Kang A. K. Patel T. R. Budhiraja S. Gaelen J. I.. (2023). Neurologic manifestations of long COVID differ based on acute COVID-19 severity. Ann. Neurol. 94, 146–159. doi: 10.1002/ana.26649, PMID: 36966460 PMC10724021

[ref39] Rofail D. Somersan-Karakaya S. Choi J. Y. Przydzial K. Zhao Y. Hussein M.. (2024). Thematic analysis to explore patients’ experiences with long COVID-19: a conceptual model of symptoms and impacts on daily lives. BMJ Open. 14:e076992. doi: 10.1136/bmjopen-2023-076992, PMID: 38233059 PMC10806796

[ref40] Romero M. Caicedo M. Díaz A. Ortega D. Llanos C. Concha A.. (2023). Post-COVID-19 syndrome: descriptive analysis based on a survivors’ cohort in Colombia. Global Epidemiol. 6:100126. doi: 10.1016/j.gloepi.2023.100126, PMID: 38023981 PMC10643089

[ref41] Sakhamuri S. M. Jankie S. Pinto Pereira L. M. (2022). Calling on Latin America and the Caribbean countries to recognise the disability from long COVID. Lancet Regional Health-Americas. 15:100362. doi: 10.1016/j.lana.2022.100362, PMID: 36043156 PMC9412073

[ref15] Santos Guedes De Sa K. Silva J. Bayarri-Olmos R. Brinda R. Alec Rath Constable R. Colom Diaz P. A.. (2024). A causal link between autoantibodies and neurological symptoms in long COVID. medRxiv. [Preprint]. doi: 10.1101/2024.06.18.24309100 PMC13501478 42208499

[ref42] Taquet M. Sillett R. Zhu L. Mendel J. Camplisson I. Dercon Q.. (2022). Neurological and psychiatric risk trajectories after SARS-CoV-2 infection: an analysis of 2-year retrospective cohort studies including 1 284 437 patients. Lancet Psychiatry. 9, 815–827. doi: 10.1016/S2215-0366(22)00260-7, PMID: 35987197 PMC9385200

[ref43] Terwee C. B. Peipert J. D. Chapman R. Lai J.-S. Terluin B. Cella D.. (2021). Minimal important change (MIC): a conceptual clarification and systematic review of MIC estimates of PROMIS measures. Qual. Life Res. 30, 2729–2754. doi: 10.1007/s11136-021-02925-y, PMID: 34247326 PMC8481206

[ref44] Toniolo S. Di Lorenzo F. Scarioni M. Frederiksen K. S. Nobili F. (2021). Is the frontal lobe the primary target of SARS-CoV-2? JAD. 81, 75–81. doi: 10.3233/JAD-210008, PMID: 33720900

[ref45] Vélez-Santamaría R. Fernández-Solana J. Méndez-López F. Domínguez-García M. González-Bernal J. J. Magallón-Botaya R.. (2023). Functionality, physical activity, fatigue and quality of life in patients with acute COVID-19 and long COVID infection. Sci. Rep. 13:19907. doi: 10.1038/s41598-023-47218-1 37963962 PMC10645778

[ref46] Visvabharathy L. Zhu C. Orban Z. Yarnoff K. Palacio N. Jimenez M.. (2023). Autoantibody production is enhanced after mild SARS-CoV-2 infection despite vaccination in individuals with and without long COVID. medRxiv. [Preprint]. doi: 10.1101/2023.04.07.23288243

[ref47] Von Elm E. Altman D. G. Egger M. Pocock S. J. Gøtzsche P. C. Vandenbroucke J. P. (2008). The strengthening the reporting of observational studies in epidemiology (STROBE) statement: guidelines for reporting observational studies. J. Clin. Epidemiol. 61, 344–349. doi: 10.1016/j.jclinepi.2007.11.008, PMID: 18313558

[ref48] Weintraub S. Dikmen S. S. Heaton R. K. Tulsky D. S. Zelazo P. D. Bauer P. J.. (2013). Cognition assessment using the NIH toolbox. Neurology. 80, S54–S64. doi: 10.1212/WNL.0b013e3182872ded, PMID: 23479546 PMC3662346

[ref37] World Health Organization. (2021). COVID-19 clinical management: living guidance, 25 January 2021. Available at: https://iris.who.int/handle/10665/338882 (Accessed April 18, 2024).

[ref49] Yang J. Markus K. Andersen K. M. Rudolph A. E. McGrath L. J. Nguyen J. L.. (2024). Definition and measurement of post-COVID-19 conditions in real-world practice: a global systematic literature review. BMJ Open. 14:e077886. doi: 10.1136/bmjopen-2023-077886, PMID: 38233057 PMC10806676

